# *Cis*-acting elements in its 3′ UTR mediate post-transcriptional regulation of *KRAS*

**DOI:** 10.18632/oncotarget.7599

**Published:** 2016-02-22

**Authors:** Minlee Kim, Nicole Kogan, Frank J. Slack

**Affiliations:** ^1^ Department of Molecular, Cellular and Developmental Biology, Yale University, New Haven, CT, USA; ^2^ Current address: Department of Biological Engineering, Massachusetts Institute of Technology, Cambridge, MA, USA; ^3^ Institute for RNA Medicine, Department of Pathology, Beth Israel Deaconess Medical Center/Harvard Medical School, Boston, MA, USA

**Keywords:** KRAS, 3′ UTR, post-transcriptional regulation, microRNAs (miRNAs), miR-185

## Abstract

Multiple RNA-binding proteins and non-coding RNAs, such as microRNAs (miRNAs), are involved in post-transcriptional gene regulation through recognition motifs in the 3′ untranslated region (UTR) of their target genes. The *KRAS* gene encodes a key signaling protein, and its messenger RNA (mRNA) contains an exceptionally long 3′ UTR; this suggests that it may be subject to a highly complex set of regulatory processes. However, 3′ UTR-dependent regulation of *KRAS* expression has not been explored in detail. Using extensive deletion and mutational analyses combined with luciferase reporter assays, we have identified inhibitory and stabilizing *cis*-acting regions within the *KRAS* 3′ UTR that may interact with miRNAs and RNA-binding proteins, such as HuR. Particularly, we have identified an AU-rich 49-nt fragment in the *KRAS* 3′ UTR that is required for *KRAS* 3′ UTR reporter repression. This element contains a miR-185 complementary element, and we show that overexpression of miR-185 represses endogenous KRAS mRNA and protein *in vitro*. In addition, we have identified another 49-nt fragment that is required to promote *KRAS* 3′ UTR reporter expression. These findings indicate that multiple *cis*-regulatory motifs in the 3′ UTR of *KRAS* finely modulate its expression, and sequence alterations within a binding motif may disrupt the precise functions of *trans*-regulatory factors, potentially leading to aberrant KRAS expression.

## INTRODUCTION

Post-transcriptional gene regulation by RNA-binding proteins (RBPs) and non-coding RNAs, such as microRNAs (miRNAs), is critical for normal eukaryotic development and physiology [1-3]. RBPs and miRNAs modulate gene expression by interacting with *cis*-acting elements in the 3′ untranslated region (UTR) of their target messenger RNAs (mRNAs) in a sequence-specific manner. Both classes of *trans*-regulatory factors can play a pleiotropic role in post-transcriptional gene regulation. For example, the 3′ UTR of an mRNA can be targeted by multiple miRNAs and RBPs in the same cell. Additionally, a single miRNA or RBP can bind to sites in multiple 3′ UTRs. The interplay between miRNAs and RBPs can add yet another layer of complexity to the gene regulation scheme. For example, the RNA-binding protein HuR (ELAVL1) can compete with a variety of miRNAs for sequence specific motifs in target mRNAs [4], or it can act cooperatively to recruit a *let-7* miRNA to repress gene expression [5]. In another example, the PUM1 RBP can alter the secondary structure of its target RNA, thereby allowing miR-221 and miR-222 to access their complementary sites [6]. In these ways, 3′ UTRs can mediate post-transcriptional gene regulation by acting as venues to coordinate interactions among various *trans*-regulatory factors and *cis*-acting 3′ UTR elements.

Alternative polyadenylation (APA) is another mechanism of gene regulation whereby the length of the 3′ UTR can be altered. This mechanism has been appreciated recently as a widespread phenomenon that leads to a diversified transcriptome [7]. Through APA, the availability of certain *cis*-acting elements can be changed, thereby leading to potential alterations in gene expression. Notably, APA has been shown to be associated with cellular proliferation and cancer [8, 9]. Many oncogenes commonly have shorter 3′ UTRs, which enables them to potentially evade the inhibitory effects of miRNAs with the result of more stable protein expression [9]. In addition, single nucleotide polymorphisms (SNPs) within the 3′ UTR also have the potential to dysregulate gene expression by introducing sequence modifications and disrupting *cis*-acting regulatory elements. SNPs have been shown to be associated with cancer risk, outcome, and drug resistance [10-12]. Therefore, alterations in the 3′ UTR sequence may lead to disruptions in gene regulation.

*KRAS*, which encodes a GTPase signaling protein, plays a major role in tumorigenesis [13]. Due to its exceptionally long 3′ UTR length, the *KRAS* gene is presumed to be regulated at the post-transcriptional level through a highly complex interaction of *cis*-acting elements within its 3′ UTR. An array of *trans*-regulatory factors - RBPs and miRNAs - known to regulate *KRAS* are often misexpressed in various types of cancer. For instance, the *KRAS*-regulating RBP, IGF2BP1 [14], is upregulated in colon cancer, while the expression of a number of miRNAs, including *let-7* [10, 15], miR-181 [16, 17], miR-96 [18], and miR-30c [19] have been shown to be downregulated in lung cancer, oral squamous carcinoma, glioma, pancreatic cancer, and breast cancer, respectively (reviewed in Kim and Slack 2014 [20]).

To date, *KRAS trans*-regulatory factors have been primarily identified through computational predictions and large-scale CLIP-seq and RNA-IP profiling of cancer cell lines and human tumor samples. However, no study that examines the post-transcriptional regulation of *KRAS* by empirically dissecting its 3′ UTR has been performed. Through extensive deletion and mutational analyses of the *KRAS* 3′ UTR, we sought to identify key *cis*-regulatory regions within the *KRAS* 3′ UTR that interact with *trans*-regulatory factors. We revealed that the *KRAS* 3′ UTR contains multiple stabilizing and inhibitory regions. The findings in this study suggest that *KRAS* is regulated through multiple *cis*-regulatory motifs in its 3′ UTR, which have the potential to interact with various RBPs and miRNAs. In particular, we identified two individual 49-nucleotide (nt) fragments that were required for robust *KRAS* 3′ UTR reporter repression and overexpression, respectively. The repressive sequence element appears to interact with miR-185, and mutations in the seed region of this repressive fragment disrupt the binding of miR-185. Thus, miR-185 appears to play a role in negatively regulating *KRAS* at the post-transcriptional level, an effect that may be achieved through cooperation with other as yet unidentified *trans*- regulatory factors.

## RESULTS

### HuR and miRNAs regulate *KRAS* through its 3′ UTR in HeLa cells

**Figure 1:**
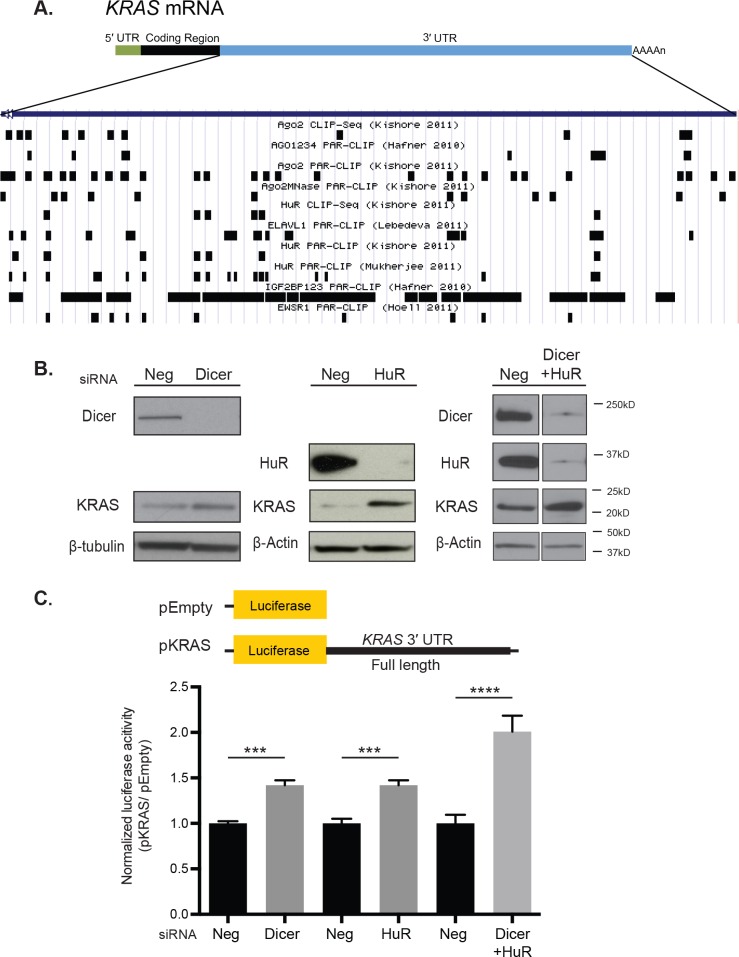
HuR and miRNAs potentially regulate *KRAS* through its 3′ UTR in HeLa cells **A.** Validated RBP binding sites from the DoRiNA database were aligned to the complete sequence of the 3′ UTR of *KRAS* transcript variant B using the UCSC Genome Browser. Only a select list of RBPs, including AGO2, HuR, IGF2BP1, 2 and 3, and EWSR1, are included in the figure. **B.** Western blot analysis showed an increase in KRAS protein level following siRNA-directed knock-down for Dicer and HuR individually and in combination in HeLa cells compared with negative control (Neg). β-tubulin and β-Actin were used as loading controls. **C.** Luciferase reporter assays showed an increase in the normalized pKRAS reporter expression with si-HuR and si-Dicer treatment compared with si-Neg treatment. pKRAS contains the full length *KRAS* 3′ UTR in the psiCHECK-2 dual luciferase vector. pEmpty is a psiCHECK-2 alone with no insert. The expression of pKRAS was normalized to that of pEmpty with each siRNA treatment. A representative of three independent experiments is shown in mean ± S.D. ***: *p*-value < 0.001, ****: *p*-value < 0.0001.

To identify RBPs and their binding motifs that may be important in regulating *KRAS*, we utilized the doRiNA database [21]. This database compiles published CLIP experiments and would allow us to align experimentally validated RBP binding motifs to the complete sequence of the 3′ UTR of *KRAS* variant B (Figure 1A). Of the many RBPs that bind to the *KRAS* 3′ UTR, AGO2 [22, 23], HuR (or ELAVL1) [4, 22, 24], IGF2BP1/2/3 [23], PUM2 [23], EWSR1 [25], TAF15 [25], FUS [25], and TIA1 [26] have been previously implicated in cancer. Among these, HuR and AGO2 were extensively validated by a variety of CLIP methods to bind to the *KRAS* 3′ UTR. HuR stabilizes its target mRNAs by binding to AU-rich elements in their 3′ UTRs, and AGO2 binds miRNAs and functions in the RNA-induced silencing complex (RISC) in miRNA-mediated regulation.

In order to determine the role of HuR and miRNAs in the regulation of *KRAS*, HeLa cells were transiently transfected with siRNAs against HuR, Dicer, and a scramble control. As Dicer is essential for miRNA biogenesis, knocking down Dicer is an effective way of examining the impact of global miRNA depletion in the cell. Western blot analysis revealed that knocking down HuR and Dicer individually increased KRAS protein levels relative to the control siRNA (Neg) (Figure 1B). To assess whether this change was mediated at the post-transcriptional level, the full 3′ UTR of *KRAS* was fused downstream of the psiCHECK-2 dual-luciferase vector (pKRAS) and its reporter expression was measured following si-HuR and si-Dicer knock down. The pKRAS expression increased following knock-down of HuR and Dicer individually in HeLa cells relative to its expression with si-Neg (Figure 1C). These findings indicate that HuR and potentially miRNAs regulate KRAS expression, at least partially through its 3′ UTR.

In addition, HuR and Dicer were knocked down in combination to determine potential cooperation or competition between HuR and miRNAs for binding sites in the *KRAS* 3′ UTR. In HeLa cells, the double knock-down revealed an increase in KRAS protein levels and a 2-fold increase in the pKRAS expression, which is an additive effect of the individual knock-down (Figure 1B and 1C). The reporter expression suggests HuR and miRNAs independently mediate *KRAS* regulation through its 3′ UTR.

### Deletion analyses identify multiple stabilizing and inhibitory regions in the *KRAS* 3′ UTR

**Figure 2:**
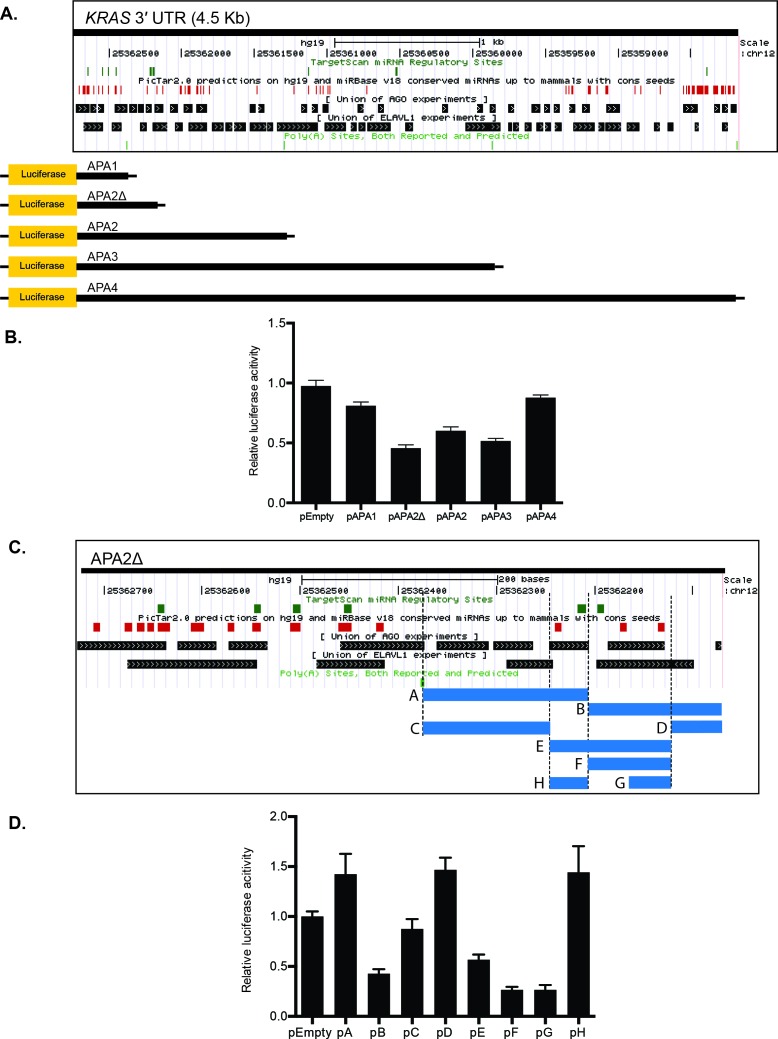
The *KRAS* 3′ UTR contains multiple stabilizing and inhibitory elements **A.** The UCSC Genome Browser was utilized to align potential miRNA binding sites, and AGO2 and HuR binding sites within the *KRAS* 3′ UTR. Truncated *KRAS* 3′ UTR luciferase reporter constructs contained varying lengths of the 3′ UTR corresponding to predicted alternative polyadenylation (APA) sites. miRNA binding sites were predicted using TargetScan and PicTar. **B.** The luciferase reporter expression of each construct was normalized to pEmpty. Luciferase reporter assays showed a trend for greater reporter repression with constructs containing longer *KRAS* 3′ UTR fragments, except for two reporters: pAPA4 and pAPA2Δ. pAPA4, which contains the full length *KRAS* 3′ UTR, showed a minimal repression, while pAPA2Δ showed the greatest repression. **C.** The 300-bp *KRAS* 3′ UTR fragment in pAPA2Δ that is not part of pAPA1 was further dissected based on the presence of potential binding sites corresponding to miRNAs and AGO2 and HuR (or ELAVL1) binding sites. miRNA predictions from TargetScan and PicTar, and the union of AGO2- and HuR- CLIP experiments from the DoRiNa database are included in the figure. These smaller fragments indicated by the blue bars were fused to psiCHECK-2 to generate 8 luciferase reporter constructs. The regulatory factors that bind to the fragment H (chr12: 25362194-25362242 in GRCh37/hg19) include miR-181 and miR-1197 predicted by PicTar and TargetScan and AGO2, FMR1, FOX2, IGF2BP1-3, and PTB predicted by DoRiNA and StarBaseV2. miRanda, miRDB, PicTar, PITA and TargetScan predict miR-29a, miR-185, miR-186, miR-548n, miR-577, miR-587 and miR-1275 binding sites and dbRBP, DoRiNA and StarBaseV2 predict AGO2, EWSR1, HuR, IGF2BP1-3, LIN28A and TTP binding sites in the fragment G (chr12: 25362099-25362147). **D.** Luciferase reporter assays revealed multiple stabilizing and inhibitory regions within the 300 bp fragment of pAPA2Δ. Of note, pH exhibited robust reporter expression, while pG exhibited a robust reporter repression compared with pEmpty. A representative of at least three independent experiments is shown in mean ± S.D. in B. and D.

Since the *KRAS* 3′ UTR provides numerous binding motifs for many RBPs and miRNAs, we employed a series of truncation analyses of the *KRAS* 3′ UTR to determine regions important for *KRAS* regulation. The first set of analyses included five separate luciferase reporters that included different lengths of the *KRAS* 3′ UTR (Figure 2A). The pAPA1, 2, 3, and 4 reporter constructs correspond to each of the four predicted polyadenylation sites for the *KRAS* 3′ UTR, as currently annotated in polyA_DB in UCSC Genome Browser [27]. The pAPA2Δ reporter corresponds to an additional polyadenylation site, which was previously listed in the human 2007 annotation in AceView [28]. Following separate transient transfection in HeLa cells of each of the five reporters or an empty vector control (pEmpty), we observed a general trend of increasing reporter repression with the longer 3′ UTR sequence constructs (Figure 2B). This might be expected, since the longer *KRAS* 3′ UTR presumably contains more regulatory elements, including potentially repressive miRNA complementary sites. Nevertheless, pAPA4, which contained the full *KRAS* 3′ UTR, exhibited the least detectable repression compared with the empty vector control under these conditions (Figure 2B). In addition, pAPA2Δ, which contained the second shortest fragment, showed the most repression among the five reporters (Figure 2B). The reporter assay suggests that potentially strong stabilizing elements exist near the 3′ end of the 3′ UTR, and multiple inhibitory and stabilizing regulatory regions reside across the entire 3′ UTR.

Interestingly, the two shortest constructs, pAPA2Δ and pAPA1, showed different capabilities for reporter repression. This suggested the existence of a potential repressive element in the 300-base pair (bp) *KRAS* 3′ UTR fragment in pAPA2Δ that did not exist in pAPA1. To examine the repressive potential of this 300-bp region, we aligned predicted miRNA complementary sites from TargetScan [29] and PicTar [30], as well as validated RBP binding motifs from the doRiNA database to the 300-bp *KRAS* 3′ UTR fragment. We then generated a series of additional reporters that contained these predicted binding motifs within the 300-bp region (Figure 2C).

When we compared each fragment with the empty vector control, we observed a robust 2-fold repression from the reporter containing the B fragment (pB) and the reporter containing the E fragment (pE), and a 3-fold repression from the reporter containing the G fragment (pG). Conversely, we observed increased expression of the reporter containing fragments A and H, which both showed a 1.5-fold increased reporter expression compared to the empty vector control (Figure 2D). Interestingly, the pE reporter construct, which included a fragment covering pG and pH, as well as their intervening 46-nt sequence, showed a 2-fold repression, suggesting that in this context the repressive element within pG can overcome a stabilizing element in pH.

### The 49-nt fragment alone in pG is required and sufficient for reporter repression

**Figure 3:**
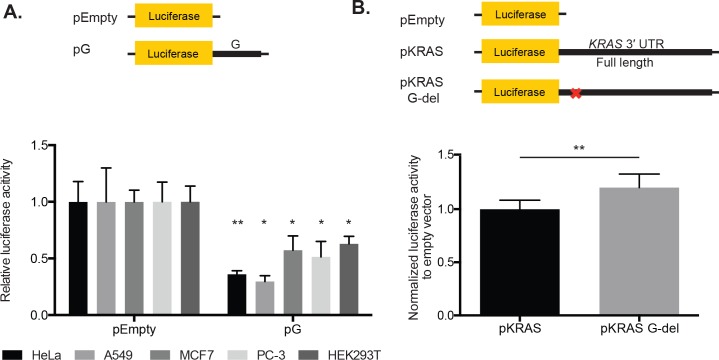
The 49-nt fragment G in the *KRAS* 3′ UTR contains a repressive element that is required for luciferase reporter repression in HeLa cells **A.** The pG reporter construct, containing the 49-nt fragment G alone, showed luciferase reporter repression in HeLa, A549, MCF7, PC-3, and HeK293T cells. Expression was normalized to pEmpty. **B.** Deletion of the fragment G sequence from the *KRAS* 3′ UTR (pKRAS G-del) resulted in a modest but statistically significant reporter de-repression compared with pKRAS, which contained the full length *KRAS* 3′ UTR. *p*-value = 0.0021. A representative of two and at least three independent experiments are shown in mean ± S.D. in A. and B. respectively. *: *p*-value <0.05, **: *p*-value <0.01.

To confirm the repression that we initially observed with the pG reporter in HeLa cells, we also examined pG expression in various human cell lines, including A549, MCF7, PC-3, and HEK293T. We observed repression, albeit to varying degrees, in all the cell lines tested, suggesting that repression of the pG reporter is not cell-type specific (Figure 3A).

To further establish whether the 49-nt fragment alone is sufficient to cause repression, we generated a reporter that contained the full *KRAS* 3′ UTR with a deletion of just the 49-nt fragment (pKRAS G-del). In HeLa cells, this deletion construct resulted in a modest but statistically significant de-repression in the reporter expression relative to a construct with no deletion (*KRAS* 3′ UTR Full length; Figure 3B). Together, our findings indicate that this small 49-nt fragment of the *KRAS* 3′ UTR is both required and sufficient for reporter repression in HeLa cells.

### The full 49-nt sequence, but not the secondary structure of the fragment in pG, is required for reporter repression

**Figure 4:**
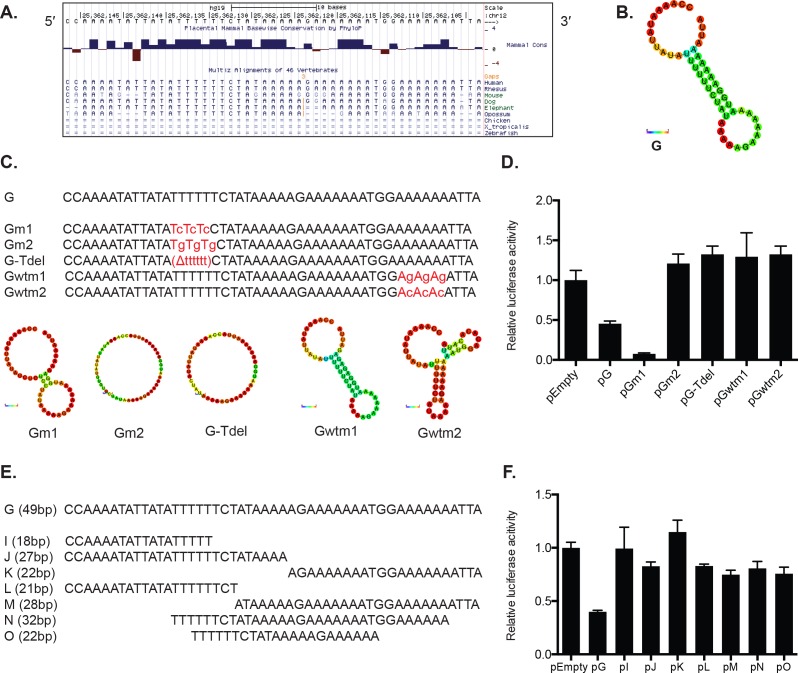
The full sequence of the 49-nt fragment G is required for luciferase reporter repression in HeLa cells **A.** A detailed survey of the 49-nt fragment G sequence revealed conserved A and U rich regions. The UCSC Genome Browser was utilized to examine the conservation across different vertebrate species. **B.** RNAfold software revealed a hairpin secondary structure for the fragment G. The color represents base-pair probabilities for each paired or unpaired bases. Blue denotes a possibility of 0 and red a possibility of 1. **C.** A series of substitution mutations were introduced within conserved A and U rich regions of the fragment G. pGm1 and pGm2 constructs were generated by mutating alternating Ts to Cs (Gm1) or Ts to Gs (Gm2). In construct pG-Tdel, the stretch of 5′ conserved Ts was deleted completely from the fragment. Gwtm1 mutated alternating As to Gs, and Gwtm2 mutated alternating As to Cs. RNAfold predicted that the original hairpin structure of fragment G was disrupted by all mutations except for the Gwtm1. **D.** Luciferase assays revealed that the T-to-G sequence mutation (pGm2), deletion of the conserved 5′ Ts (pG-Tdel), and mutations of the conserved 3′ As (pGwtm1 and pGwtm2) resulted in de-repression compared to the original G sequence. The T-to-C sequence mutation (pGm1) exhibited enhanced repression compared with unmutated fragment G (pG). **E.** Further truncation of the fragment G was performed to generate seven additional smaller fragments that were cloned into psiCHECK-2. **F.** All reporters containing the new smaller fragments exhibited a relief of the reporter repression initially observed in pG. A representative of at least three independent experiments is shown in mean ± S.D. in D. and F.

A detailed survey of the 49-nt fragment in the pG construct revealed a conserved AU-rich element (ARE) near its 5′ end (Figure 4A). RNAfold [31] predicts a secondary hairpin structure that contains a stem between the 5′ ARE and a string of thymines near the 3′ end (Figure 4B).

To test the functional significance of the ARE and the secondary hairpin structure of the 49-nt fragment in reporter repression, we first created constructs that included either deletion or substitution mutations within the ARE (Figure 4C). In two constructs, pGm1 and pGm2, the stretch of six thymines in the ARE sequence were alternatively substituted with cytosines or guanines. An additional construct, pG-Tdel, deleted the entire six-thymine stretch. We observed that reporters containing the T deletion (pG-Tdel) and the T-to-G substitution mutation (pGm2) resulted in a relief of repression compared to a reporter without any nucleotide changes in the 49-nt fragment (reporter pG; Figure 4D). Surprisingly, the T-to-C substitution mutation (pGm1) yielded a greater repression compared to the reporter pG (Figure 4D). Differential reporter expression observed by these mutation analyses suggests that the A- and T-rich regions of the *KRAS* 3′ UTR are indeed important for the reporter repression, and it is possible that these regions might function as motifs for binding of a repressive *trans*-regulatory factor.

In addition, two more reporters - pGwtm1 and pGwtm2 - were designed to disrupt the hairpin structure by introducing G or C substitution mutations in the stretches of adenosines on the 3′ end of the predicted hairpin stem (Figure 4C). Interestingly, pGwtm1 was predicted to maintain the original secondary structure due to the G-U wobble base pairing (Figure 4C). However, both reporters showed de-repression compared to pG (Figure 4D). We also observed very minimal or no repression compared with pG when we looked at seven additional reporters containing truncated portions of the 49-nt fragment (Figure 4E and 4F). Altogether, these data suggest that the full, intact 49-nt sequence, but not the secondary structure, is required for the observed reporter repression.

### miR-185 regulates the pG reporter and endogenous KRAS

**Figure 5:**
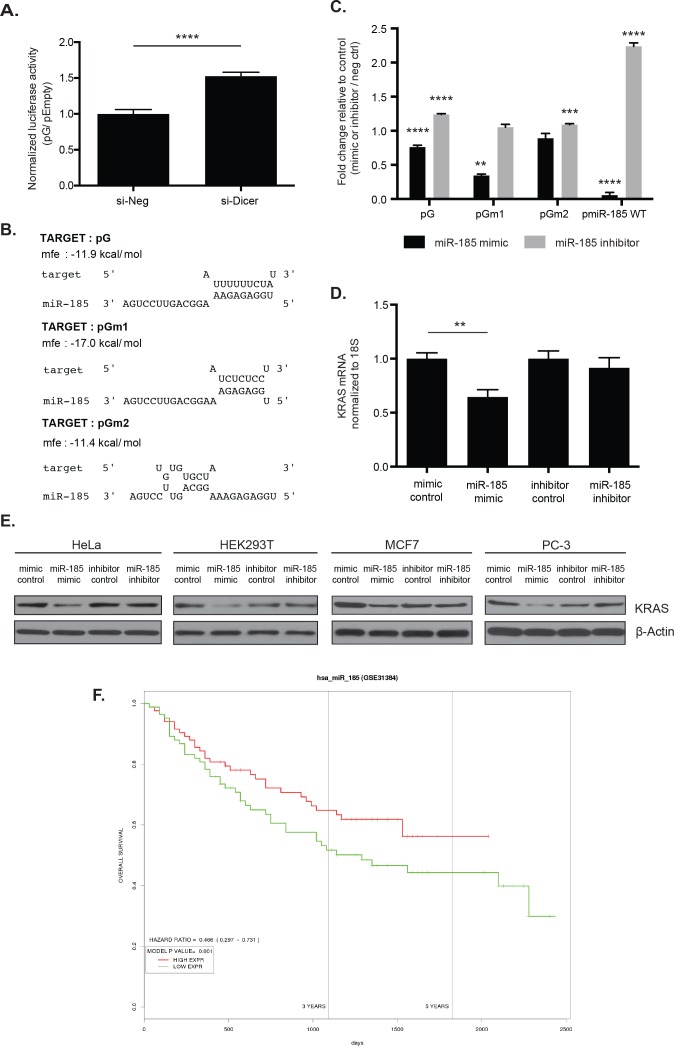
miR-185 potentially regulates KRAS through complementary sites within the 49-nt pG fragment **A.** The pG reporter construct showed a 1.5-fold luciferase reporter de-repression in HeLa cells with global inhibition of miRNA production by Dicer knock-down (si-Dicer) compared to the control siRNA (si-Neg). ****: *p*-value < 0.0001. **B.** The RNAhybrid tool predicted sequence complementarity between the seed region of miR-185 and the unmutated (G) and a T-to-C substitution mutated 49-nt fragment (Gm1). This seed region binding was abolished with a T-to-G substitution mutation (Gm2). Of note, the minimum free energy to form an RNA duplex between miR-185 and Gm1 was stronger than between miR-185 and G. **C.** In HeLa cells, luciferase assays showed enhanced repression with reporters pG and pGm1 with miR-185 mimic compared with the mimic control. miR-185 inhibitor induced a slight de-repression of pG and pGm2 reporter expression, compared with the inhibitor control. Overexpression and depletion of miR-185 by miR-185 mimic and inhibitor, respectively, were confirmed in HeLa cells with pmiR-185WT, which contained a perfect complementary sequence of miR-185. miR-185 mimic induced significant repression of pmiR-185WT, while de-repression was observed with miR-185 inhibitor compared with the respective controls. **: *p*-value < 0.01; ***: *p*-value < 0.001; ****: *p*-value < 0.0001. **D.** Total RNA from HeLa cells was analyzed for KRAS mRNA level 48hrs post-miR-185 mimic or inhibitor transfection. A 35% decrease in KRAS mRNA expression was observed following miR-185 mimic transfection compared to mimic control while no change with miR-185 inhibitor. **E.** Total cell lysates from HeLa, HEK293T, MCF7 and PC-3 were analyzed for KRAS protein level 48hrs or 72hrs post-transfection of miR-185 mimic or inhibitor. A decrease in KRAS protein expression was observed following miR-185 mimic administration compared to mimic control in these cell lines. No change in KRAS protein levels was observed with miR-185 inhibitor. β-Actin was used as a loading control. **F.** The PROGmiR tool was utilized to identify a correlation between miR-185 expression and overall survival in 16 different types of cancer. High miR-185 expression was correlated with increased overall survival only in patients with liver cancer. A representative of two independent experiments is shown in mean ± S.D. in A. and D. and of three independent experiments in C.

In order to uncover the possible *trans*-acting mechanism of *KRAS* 3′ UTR-mediated regulation, we first examined the role of miRNAs in the luciferase reporter assay. Transiently knocking down Dicer in HeLa cells, which inhibits global miRNA production in the cell, resulted in a 1.5-fold increase in pG expression compared to the negative control siRNA (Figure 5A). The change in the reporter expression suggests that miRNAs may play at least a partial role in the repression of pG.

Since the substitution mutations within the A- and T-rich regions of the G fragment of *KRAS* 3′ UTR fragment led to a change in the reporter expression, we sought to identify miRNAs that might bind within this fragment sequence. We used miRanda [32], TargetScan [29], miRDB [33], and PITA [34] to search for potential miRNA binding sites (Supplementary Figure 1A). Among the potential interacting miRNAs, miR-185 especially stood out, since it was predicted to have sequence complementarity in its seed region with the 49-nt fragment, as predicted in RNAhybrid [35] (Figure 5B). In fact, the minimum free energy required to form an RNA duplex between miR-185 and the fragment indicates that binding of miR-185 is stronger with the sequence of pGm1 than with the sequence of pG (Figure 5B). In addition, the seed region binding is abolished between miR-185 and the sequence of pGm2. Assuming that miR-185 acts as a repressing factor, these binding predictions agree with our observed reporter expression findings (Figure 4C and 4D), where we found pGm1 resulted in greater repression and pGm2 resulted in de-repression compared with pG.

The expression of miR-185 was assessed in HeLa, A549, MCF7, PC-3, and HEK293T cell lines, along with other miRNAs that were predicted to have their complementary sites within the G fragment (Supplementary Figure 1). Compared with *let-7* and miR-186, which show high levels of expression in these cell lines, miR-185 appears to be expressed at a relatively low level, although a precise quantitative comparison between the miRNAs would be required to directly compare their expression.

To determine the role of miR-185 in the *KRAS* 3′ UTR reporter expression assay, three individual reporters - pG, pGm1, and pGm2 - were transiently co-transfected with a miR-185 mimic or miR-185 inhibitor in HeLa cells. Transfecting pG and pGm1 individually with miR-185 mimic resulted in enhanced repression compared with a mimic control (Figure 5C). In contrast, transfecting pG with miR-185 inhibitor resulted in de-repression compared to the pG expression with inhibitor control (Figure 5C). We also observed a modest but statistically significant de-repression in pGm2 with miR-185 inhibitor, which we cannot explain now. To confirm the transfection efficiency of miR-185 mimic and inhibitor, we created pmiR-185WT, which contained a perfect complementary sequence of miR-185 in the same luciferase vector used for our *KRAS* 3′ UTR reporter assays. We observed a robust repression in pmiR-185WT with miR-185 mimic and de-repression with miR-185 inhibitor, which confirmed the overexpression and depletion of miR-185, respectively, in the cells (Figure 5C).

In addition, miR-185 mimic and inhibitor were individually transfected into HeLa cells in order to assess the effect of miR-185 on KRAS mRNA and protein levels. As observed with the pG reporter, overexpression of miR-185 with the miR-185 mimic led to a decrease in KRAS mRNA and protein levels compared to its control (Figure 5D and 5E). However, inhibition of miR-185 did not result in a noticeable change compared with the control possibly due to the low endogenous levels of miR-185 (Figure 5D and 5E). The decrease in KRAS protein levels with miR-185 mimic was confirmed in other cell lines, including HEK293T, MCF7, and PC-3 (Figure 5E). These findings indicate that miR-185 interacts with its complementary sites, such as one in the 49-nt fragment of the *KRAS* 3′ UTR, to regulate mRNA stability and translation of *KRAS*.

We also examined the correlation between miR-185 and overall survival using PROGmiR [36], a tool that compiled data from TCGA and GEO to study the prognostics of miRNAs in 16 different types of cancer. We found that low miR-185 expression is correlated with poor prognosis for liver cancer (Figure 5F), while high miR-185 expression is correlated with poor prognosis for head and neck cancer, acute myeloid leukemia, and renal cancer (Supplementary Figure 2). The results from PROGmiR suggest that miR-185 functions in a cell type- and tissue-specific manner.

### Additional possible trans-acting RNAs

In addition to miRNAs, it is possible that the reporter repression observed with the pG 49-nt fragment is achieved through other non-coding RNA mechanisms, such as long non-coding RNAs (lncRNAs). A predicted lncRNA (LOC101928562), identified from a BLAT search, was found to form a perfect 20-nt base paring with a portion of the fragment G by RNAhybrid. However, we were not able to detect the expression of this lncRNA in HeLa cells to successfully clone it into a plasmid. This was possibly due to the gender or cell specificity of this particular lncRNA, which was originally identified from a cDNA clone from adult testis [37] (data not shown), suggesting that this lncRNA was not responsible for repression mediated through the G fragment.

## DISCUSSION

In this study, we aimed to empirically determine the key regulatory regions within the long 3′ UTR of *KRAS*. Through a series of truncations and deletions of the *KRAS* 3′ UTR, we found that the *KRAS* 3′ UTR features multiple repressive and stabilizing *cis*-acting regions with which numerous *trans*-regulatory factors can potentially interact. Notably, the 3′ UTR appears to contain a strong stabilizing region near its 3′ end (Figure 2B). This observation is somewhat contrary to the widespread phenomenon that cancer-associated genes tend to have a shorter 3′ UTR in order to yield more stabilized proteins [9]. Nevertheless, we can speculate that in the case of *KRAS* in HeLa cells, having a longer 3′ UTR may be more advantageous for cellular proliferation by providing several binding motifs for mRNA stabilizing *trans*-regulatory factors. In addition, the full sequence of the *KRAS* 3′ UTR may achieve a more stabilized secondary structure compared with the shorter isoform.

We identified a 49-nt *cis*-regulatory region of the *KRAS* 3′ UTR, fragment G, which is both necessary and sufficient for reporter repression in HeLa cells. Truncation analyses of this 49-nt region revealed that the sequence, though probably not the structure, is required for its observed repression in luciferase reporter assays in various cell lines. We also identified a second 49-nt fragment of the *KRAS* 3′ UTR - fragment H - which contained potential stabilizing elements; this will be interesting to examine in future studies.

To elucidate the possible mechanisms by which the *KRAS* 3′ UTR reporter was regulated, we examined the role of non-coding RNAs, including miRNAs and long non-coding RNAs (lncRNAs). While our investigation of lncRNAs was inconclusive, we found that miRNAs play at least a partial role in mediating repression of the 49-nt pG reporter. Specifically, we observed that knocking down Dicer in HeLa cells resulted in a 1.5-fold increase in the expression of the pG reporter construct compared with a control siRNA. Dicer depletion also led to an increase in KRAS protein.

By utilizing several target prediction algorithms, we further identified miR-185 as a *trans*-acting factor with the potential to bind to the pG containing 49-nt regulatory region within the *KRAS* 3′ UTR. Additionally, we observed that miR-185 is needed in part for *KRAS* 3′ UTR-mediated repression in cellular reporter assays, as well as being required for repression of endogenous KRAS mRNA and protein in HeLa cells. Ectopic over-expression of miR-185 resulted in enhanced reporter repression in pG and pGm1, and depletion of miR-185 resulted in a slight de-repression only in pG. No de-repression was observed when pGm1 was transfected with miR-185 inhibitor, possibly owing to a stronger affinity between pGm1 and endogenous miR-185 than between the inhibitor and miR-185. Although miR-185 appears to affect the reporter repression, depleting miR-185 alone did not result in a full relief of repression. This is not unexpected, since knocking down an individual miRNA often does not result in phenotypic changes [38], and a combination of miRNAs may cooperate for the full reporter repression.

Previous studies have demonstrated cooperative activity of RPBs and miRNAs for regulating target RNA expression. For example, binding of the PUM1 protein to the 3′ UTR of *p27* alters the structure of the *p27* mRNA and exposes functional binding sites for miR-221 and miR-222 [6]. Our findings, which showed that knocking down HuR and Dicer individually increased KRAS protein levels relative to the control siRNA, led us to speculate that cooperation between RBPs and miRNAs might also be a potential mechanism for the repression we observed in the *KRAS* 3′ UTR reporter assays. Therefore, we examined the possibility of a cooperative role of miR-185 and candidate RBPs (IGF2BP1, IGF2BP2, IGF2BP3, EWSR1, and HuR), which we selected from a database search using two CLIP databases, DoRiNA [21] and StarBase V2 [39]. However, none of the candidate RBPs tested were found to yield pG reporter de-repression in HeLa cells following knock-down of the individual candidate RBP genes, along with a miR-185 inhibitor (data not shown).

We speculate that two potential issues may account for this lack of differential reporter expression. First, since those RBPs were identified in CLIP experiments performed in HEK293 cells, the miRNAs and RBPs used in our assay system may be differentially expressed in HeLa cells, the cells in which the majority of our reporter experiments were performed. Secondly, during CLIP experiments, overexpression of proteins creates an artificial condition, which may lead to some cases of aberrant interactions, as well as disrupt natural physiological interactions between endogenous mRNAs and RBPs. However, more direct biochemical approaches, such as RNA-protein complex pull-down followed by mass spectrometry, may provide more informative evidence concerning which RBPs bind to the 49-nt 3′ UTR fragment.

miR-185 is a known tumor-suppressive miRNA that has been shown to inhibit proliferation in HeLa cells [40]. We speculate that miR-185 could also play a potential role in regulating *KRAS* based on our western blot data showing that miR-185 affects KRAS protein expression in various cell lines (Figure 5E). miR-185 has clinical relevance and has been reported to be deregulated in various cancers, including lung cancer, glioma, hepatocellular carcinoma, gastric cancer, and breast cancer [41-46]. By targeting DNMT1, RhoA/Cdc42, and E2F6, miR-185 has been shown to induce cell cycle arrest and apoptosis, in addition to inhibiting proliferation and invasion in cancer cell lines and xenograft mouse models of various cancers [40, 44, 46]. Furthermore, low miR-185 expression has been correlated with reduced overall survival and relapsed-free survival in gastric cancer [43] and triple-negative breast cancer [44]. In our own analysis using PROGmiR [36], we found that low miR-185 expression is correlated with poor prognosis for liver cancer (Figure 5F). In support of this, an independent study by Zhi et al. revealed that that miR-185 can be a prognostic tool of early stage hepatocellular carcinoma for survival and recurrence [47]. In contrast, our analysis using PROGmiR indicated that high miR-185 expression is correlated with poor prognosis for head and neck cancer, acute myeloid leukemia, and renal cancer (Supplementary Figure 2). As reported previously, some miRNAs have two opposing roles depending on the cellular context, and it appears that miR-185 may also fall into this category of miRNAs.

Together, our findings provide evidence for the presence of multiple inhibitory and stabilizing *cis*-acting elements within the *KRAS* 3′ UTR. Two of these elements represent individual sequence fragments with the potential to interact with post-transcriptional regulatory factors, including miRNAs and RBPs. We identified the tumor suppressive miRNA, miR-185, to interact with the *KRAS* 3′ UTR via a 49-nt fragment and possibly via other regions as well, such as one miR-185 binding site about 500 bp away from the end of the 3′ UTR predicted by TargetScan. Interestingly, within the repressive 49-nt fragment, a SNP, rs547078411, resides at the first nucleotide of the predicted miR-185 target site, and a T-to-C somatic mutation was identified at chr12: 25362140 (Hg19) in a lung cancer study (COSU583) in the COSMIC database. The potential role of these nucleotide changes in tumorigenesis remains to be determined. Further exploration to determine how multiple *cis*- and *trans*-regulatory factors collectively cooperate to regulate *KRAS* will provide crucial insights into the 3′ UTR-dependent regulation of *KRAS* and will allow a more profound understanding of the mechanisms involved in *KRAS*-associated tumorigenesis.

## MATERIALS AND METHODS

### Generation of the *KRAS* 3′ UTR luciferase reporters

To generate luciferase reporters with varying lengths of the *KRAS* 3′ UTR, the construct, pGL4.75 KRAS#13 mLCS1, which was previously generated by Dr. Lena J. Chin [10], was used as a template. This template contains a 3910 bp region of the *KRAS* 3′ UTR originally cloned from DNA isolated from human genomic DNA. To generate the full-length *KRAS* 3′ UTR vector (pKRAS), we amplified the remaining 671-nt from the 3′ end of the 3′ UTR of *KRAS* separately from HeLa genomic DNA and then annealed it to the pGL4.75 KRAS#13 mLCS1 template using overlapping PCR with the Expand High Fidelity PCR System (Roche) and the primers listed below (Table 1). 3′ UTR truncation constructs were generated from the pKRAS vector using the primers listed below (Table 1). Each 3′ UTR fragment was amplified with Phusion High-Fidelity DNA polymerase (NEB), and cloned into the *Xho*I and *Not*I sites in the dual-luciferase vector, psiCHECK-2 (Promega). Deletions and mutations of the *KRAS* 3′ UTR fragment were created using PCR-mediated deletion as described in Hansson *et al*. [48], and site-directed mutagenesis using an XL Site-Directed Mutagenesis Kit (Agilent) and the primers listed below (Table 1). Each construct was confirmed by sequencing using the primers listed in Table 2.

**Table 1:** Cloning primers used to construct the *KRAS* 3′ UTR reporters

Construct (insert size in bp)	Genomic position in chr12 (GRCh37/hg19)	Primer	Sequence (5′-3′)
pAPA1 (354)	25362375 - 25362728	MK1	CCCGCTCGAGATACAATTTGTACTTTTTTCTTAAGGCATAC
		MK75	ATAAGAATGCGGCCGCGGGATGATTCAAAAGCTTCATTAATTTG
pAPA2Δ (657)	25362072-25362728	MK1	CCCGCTCGAGATACAATTTGTACTTTTTTCTTAAGGCATAC
		MK2	ATAAGAATGCGGCCGCGGCCTTATAATAGTTTCCATTGCCTTG
pAPA2 (1478)	25361251-25362728	MK1	CCCGCTCGAGATACAATTTGTAC TTTTTTCTTAAGGCATAC
		MK3	ATAAGAATGCGGCCGCGCCATCTCACTTCATTTATTTTAAAATAAG
pAPA3 (2896)	25359833-25362728	MK1	CCCGCTCGAGATACAATTTGTACTTTTTTCTTAAGGCATAC
		MK4	ATAAGAATGCGGCCGCAATTGTCCTAAAAGAATCACAGTTATGC
pAPA4 or pKRAS (4583)	25358146-25362728	MK1	CCCGCTCGAGATACAATTTGTACTTTTTTCTTAAGGCATAC
		MK39	CATTTTATGACAGCTATTCAGTTTCTCAATGCA GAATTCATGCTATCCAG
		MK40	GAAACTGAATAGCTGTCATAAAATG
		MK38	ATAAGAATGCGGCCGCCAGTTCAAATTTCATGAATAAATACACACTC
pA (181)	25362194 - 25362375	MK81	CCCGCTCGAGTATTCTGTGTTTTATCTAGTCACATAAATG
		MK84	ATAAGAATGCGGCCGCGTGAACAGTGTAACTTTACATTCATC
pB (122)	25362072-25362193	MK85	CCCGCTCGAGAAA GGT TTT GTC TCC TTT CCA CTG
		MK2	ATAAGAATGCGGCCGCGGCCTTATAATAGTTTCCATTGCCTTG
pC (132)	25362243-25362374	MK81	CCCGCTCGAGTATTCTGTGTTTTATCTAGTCACATAAATG
		MK82	ATAAGAATGCGGCCGCGATGCCTAGAAGAATCATCATCAG
pD (27)	25362072-25362098	MK88	CAGTAATTCTAGGCGATCGCCAAGGCAATGGAAAC
		MK89	TAATAGTTTCCATTGCCTTGGCGATCGCCTAGAATTAC
pF (95)	25362099-25362193	MK86	GAAAAAAATGGAAAAAAATTACGGCCGCTGGCCGC
		MK87	ATTGCGGCCAGCGGCCGTAATTTTTTTCCATTTTTTTC
pG (49)	25362099-25362147	MK92	CAGTAATTCTAGGCGATCGCCCAAAATATTATATTTTTTC
		MK93	GAAAAAATATAATATTTTGGGCGATCGCCTAGAATTAC
pE (144)	25362099-25362242	NK 1f both	CCCGCTCGAGATGTCCTATAGTTTGTCATCC
		NK1r	ATAAGAATGCGGCCGCTAATTTTTTTCCATTTTTTTCTTTTTATAG
pH (49)	25362194-25362242	NK 1f both	CCCGCTCGAGATGTCCTATAGTTTGTCATCC
		MK84	ATAAGAATGCGGCCGCGTGAACAGTGTAACTTTACATTCATC
pG-Tdel (43)	N/A	NK t del f	GATCGCCCAAAATATTATACTATAAAAAGAAAAAAATGG
		NK t del r	CCATTTTTTTCTTTTTATAGTATAATATTTTGGGCGATC
pGm1 (49)	N/A	MK94	GATCGCCCAAAATATTATAtctctcCTATAAAAAGAAAAAAATGG
		MK95	CCATTTTTTTCTTTTTATAGgagagaTATAATATTTTGGGCGATC
pGm2 (49)	N/A	MK96	GATCGCCCAAAATATTATAtgtgtgCTATAAAAAGAAAAAAATGG
		MK97	CCATTTTTTTCTTTTTATAGcacacaTATAATATTTTGGGCGATC
pGwt1m (49)	N/A	NK wt 1f	CTATAAAAAGAAAAAAATGGAGAGAGATTACGGCCGCTG
		NK wt 1r	CAGCGGCCGTAATCTCTCTCCATTTTTTTCTTTTTATAG
pGwt2m (49)	N/A	NK wt 2f	CTATAAAAAGAAAAAAATGGACACACATTACGGCCGCTG
		NK wt 2r	CAGCGGCCGTAATGTGTGTCCATTTTTTTCTTTTTATAG
pKRAS G-del (4534)	N/A	MK128	AGTCATGGTCACTCTCCCAAGGCAATGGAAACTATTATAAGG
		MK129	CCTTATAATAGTTTCCATTGCCTTGGGAGAGTGACCATGACT
pI (18)	25362130-25362147	MK104	GATCGCCCAAAATATTATATTTTTCGGCCGCTGG
		MK105	TGCGGCCAGCGGCCGAAAAATATAATATTTTGG
pJ (27)	25362121-25362147	MK106	CCAAAATATTATATTTTTTCTATAAAACGGCCGCTGG
		MK107	TGCGGCCAGCGGCCGTTTTATAGAAAAAATATAATATTTTGG
pK (22)	25362099-25362121	MK108	GTAATTCTAGGCGATCGCAGAAAAAAATGGAAAAAAATTACG
		MK109	CGTAATTTTTTTCCATTTTTTTCTGCGATCGCCTAGAATTAC
pL (21)	25362147-25362128	MK116	TCGCCCAAAATATTATATTTTTTCTCGGCCGCTGG
		MK117	TGCGGCCAGCGGCCGAGAAAAAATATAATATTTTG
pM (28)	25362128-25362099	MK118	TCTAGGCGATCGCATAAAAAGAAAAAAATGGAAAAAAATTAC
		MK119	GTAATTTTTTTCCATTTTTTTCTTTTTATGCGATCGCCTAGA
pN (32)	25362136-25363103	MK120	TTTTTTCTATAAAAAGAAAAAAATGGAAAAAACGGCCGCTGGCCGCA
		MK121	TTTTTTCCATTTTTTTCTTTTTATAGAAAAAAGCGATCGCCTAGAATTACTGC
pH (22)	25362136-25362113	MK122	TTTTTTCTATAAAAAGAAAAAACGGCCGCTGGCCGCA
		MK123	TTTTTTCTTTTTATAGAAAAAAGCGATCGCCTAGAATTACTGC

**Table 2:** Sequencing primers used to confirm the *KRAS* 3′ UTR reporters

Primer	Sequence (5′-3′)
MK5	TGCTTTTGTTTCTTAAGAAAACAAACTC
MK7	TACCAGATGCCAGTCACCGCAC
MK18	GGAG GACGCTCCAG ATGAAATG
MK27	CGAGGTCCGAAGACTCATTTAGATC
LJC1	GGCACACCACCACCCCAAAATCTC
LJC3	GGGTCGTATACCAAAGGCCTTAG
LCJ5	CTAGCTAGCTCAATGCAGAATTCATGCTATCCAG

### Cell culture and *KRAS* 3′ UTR luciferase reporter assays

A549, HeLa, MCF7, and PC-3 cells were cultured in RPMI (Gibco); HEK293T cells were cultured in DMEM (Gibco). All cell cultures were supplemented with 10% FBS (Cellgro or Sigma-Aldrich) and 1X penicillin/streptomycin (Gibco). Transient DNA transfection was done using Lipofectamine 2000 (Invitrogen) and Opti-MEM (Gibco); 2 or 5 ng of purified reporter DNA was transfected into HeLa, 15 ng into A549, 15 ng into PC-3, 5 ng into MCF7, and 5 ng into HEK293T. Renilla and Firefly luciferase activities were measured at 24 hrs post-transfection using the Dual-Luciferase Reporter Assay System (Promega) and Wallac Victor 1420^2^ (Perkin Elmer) or GloMax-Multi Detection System (Promega). Two-tailed *t* tests were used to measure statistical significance of differences in reporter expression.

### Western blotting

Cells were lysed in RIPA buffer (1X PBS, 0.4% Sodium deoxycholate, 1% NP-40, 0.1% SDS) with 1X cOmplete EDTA-free Protease Inhibitor Cocktail (Roche), and protein quantity was determined using Bio-Rad or Pierce protein assays. Gel electrophoresis was performed in 1X running buffer (Bio-Rad) for Criterion XT Precast Gels (Bio-Rad) or in 1X MOPS buffer (Life Technologies) for NuPAGE Novex Bis-Tris Midi Protein Gels (Life Technologies), followed by a wet transfer per manufacturer's instruction. Blocking and antibody dilutions were performed in 5% milk in 1X TBST. Protein was detected using SuperSignal West Dura or SuperSignal West Pico Chemiluminescent Substrate (Pierce). Primary antibodies included: KRAS (F234, Santa Cruz), Dicer (H-212, Santa Cruz), β-tubulin (T4026, Sigma), GAPDH (2118, Cell Signaling), and β-Actin (C4, Santa Cruz or 691001, MP). Secondary antibodies included: goat anti-mouse IgG-HRP (sc-2031, Santa Cruz), and goat anti-rabbit IgG-HRP (sc-2004, Santa Cruz).

### miRNA mimic and inhibitor transfection and mRNA and miRNA detection

50nM of miRNA inhibitor, miRNA mimic or the corresponding Negative Control #1 (Life Technologies) was transiently transfected to the cells using DharmaFECT 1 (GE Dharmacon) or Lipofectamine RNAiMAX (Thermo Fisher Scientific). Co-transfection of miRNA inhibitor or mimic and luciferase reporter was performed using DharmaFECT Duo (GE Dharmacon). Depletion or overexpression of miR-185 was confirmed using a miRNA sensor reporter, pmiR-185WT, which was generated using primers 185wtF (5′ - TCGAGTCAGGAACTGCCTTTCTCTCCAGC - 3′) and 185wtR (5′ - GGCCGCTGGAGAGAAAGGCAGTTCCTGAC - 3′).

Cellular miRNA expression was assessed using the miRNeasy Mini Kit (Qiagen), miScript II RT (Qiagen), miScript SYBR Green kits (Qiagen), and miScript primer assays (Qiagen) in LightCycler 480 (Roche). Total RNA was extracted using the RNeasy Plus Mini Kit (Qiagen) and was reverse transcribed using SuperScript III Reverse Transcriptase following the manufacture's instruction (Invitrogen) with Oligo(dT). qPCR was performed using the primers listed (Table 3) and LightCycler^®^ 480 SYBR Green I Master mix (Roche).

**Table 3:** qPCR primers used to detect mRNA levels

Gene	Sequence (5′-3′)
HuR	AGCAGGACACAGCTTGGGCTATG
	TCGGGCGAGCATACGACACCTTAATG
AGO2	CTAACCTACCAGCTGTGTCAC
	CCTTCAGCACTGTCATGTTCC
KRAS	GACTGAATATAAACTTGTGGTAGTTGG
	CCTCTATTGTTGGATCATATTCGTC
Dicer	AGCCACTGCTGGATGTGGAC
	GAACCAGTATCTGTTTATTCTGCAG
TTP	CACTGTGGTCTCTGCATGGAC
	CACCATCATGAATACTGAGCTTG
EWSR1	CGTCCACGGATTACAGTAC
	CATATGCCTGGGTGGTCTG
IGF2BP1	CATCTCCTCGTTGCAAGACC
	TGAGACTGCAGGCTCATGG
IGF2BP2	GAGACCCTCTCGGGTAAAGTG
	CATCCAACACCTCCCACTGC
IGF2BP3	CAGTGGGAGGTGCTGGATAG
	GTCTAGTGCTTGTCTAGCTTGG
GAPDH	TGCACCACCAACTGCTTAGC
	GGCATGGACTGTGGTCATGAG
S18	CAGAATCCACGCCAGTACAAGATC
	GAGCTTGTTGTCCAGACCATTGG

## SUPPLEMENTARY MATERIAL


