# Supplementary material for: Cis-acting elements in its 3′ UTR mediate post-transcriptional regulation of KRAS
Source: Oncotarget. 2016 Feb 22;7(11):11770–84. doi: 10.18632/oncotarget.7599 (PMC4914247; doi:10.18632/oncotarget.7599)
Supplement: Supplementary file 1 [file oncotarget-07-11770-s001.pdf]

Cis-acting elements in its 3′ UTR mediate post-transcriptional regulation of *KRAS*

Supplementary Material

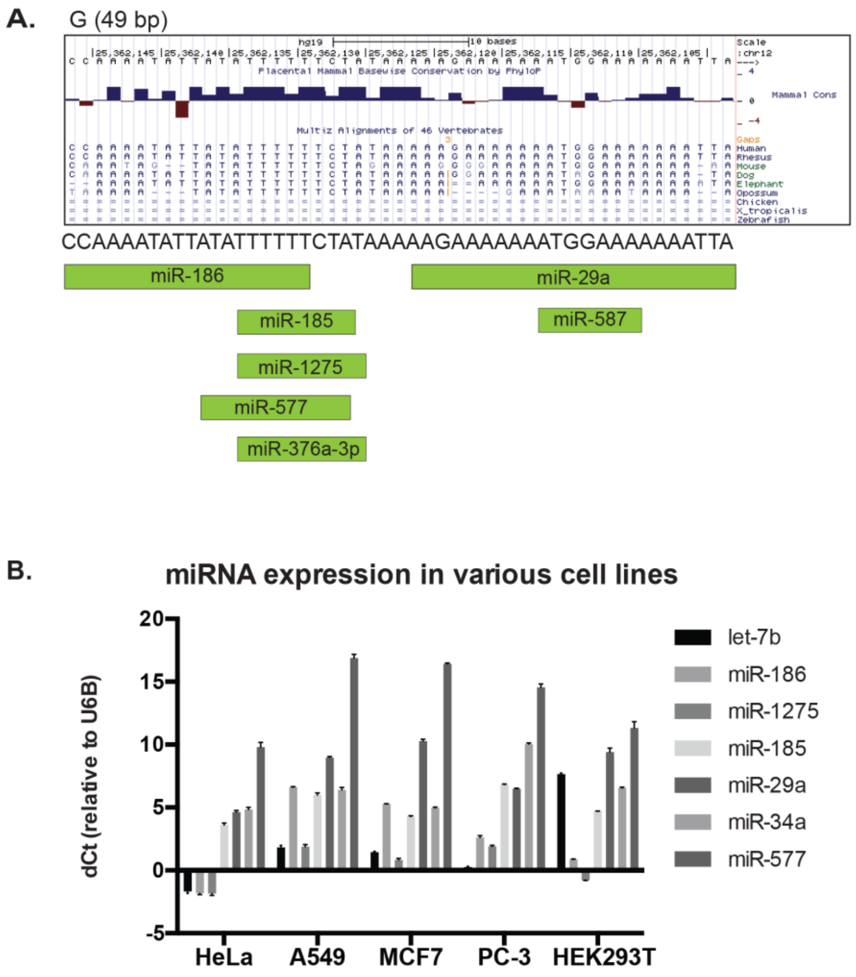

Supplementary Figure 1

A. miRanda, TargetScan, miRDB, and PITA were used to search for potential miRNA binding sites in the G fragment of the *KRAS* 3′ UTR. Since the substitution mutations within the A- and T-rich regions of the G fragment led to a change in the reporter expression, miRNAs that specifically bind to this fragment sequence were selected as potential candidates. **B.** miRNA expression was examined across different cells lines, including HeLa, A549, MCF7, PC-3, and HEK293T, using Qiagen primers specific to five miRNA candidates that were identified from

target prediction tools. *let-7b* and miR-34a were included as relative controls for miRNA expression. *let-7b* is known to be highly expressed in HeLa cells, while miR-34a is expressed at low levels in HeLa cells. Delta Ct values were used to represent the y-axis due to a lack of proper control cell line with which to normalize.

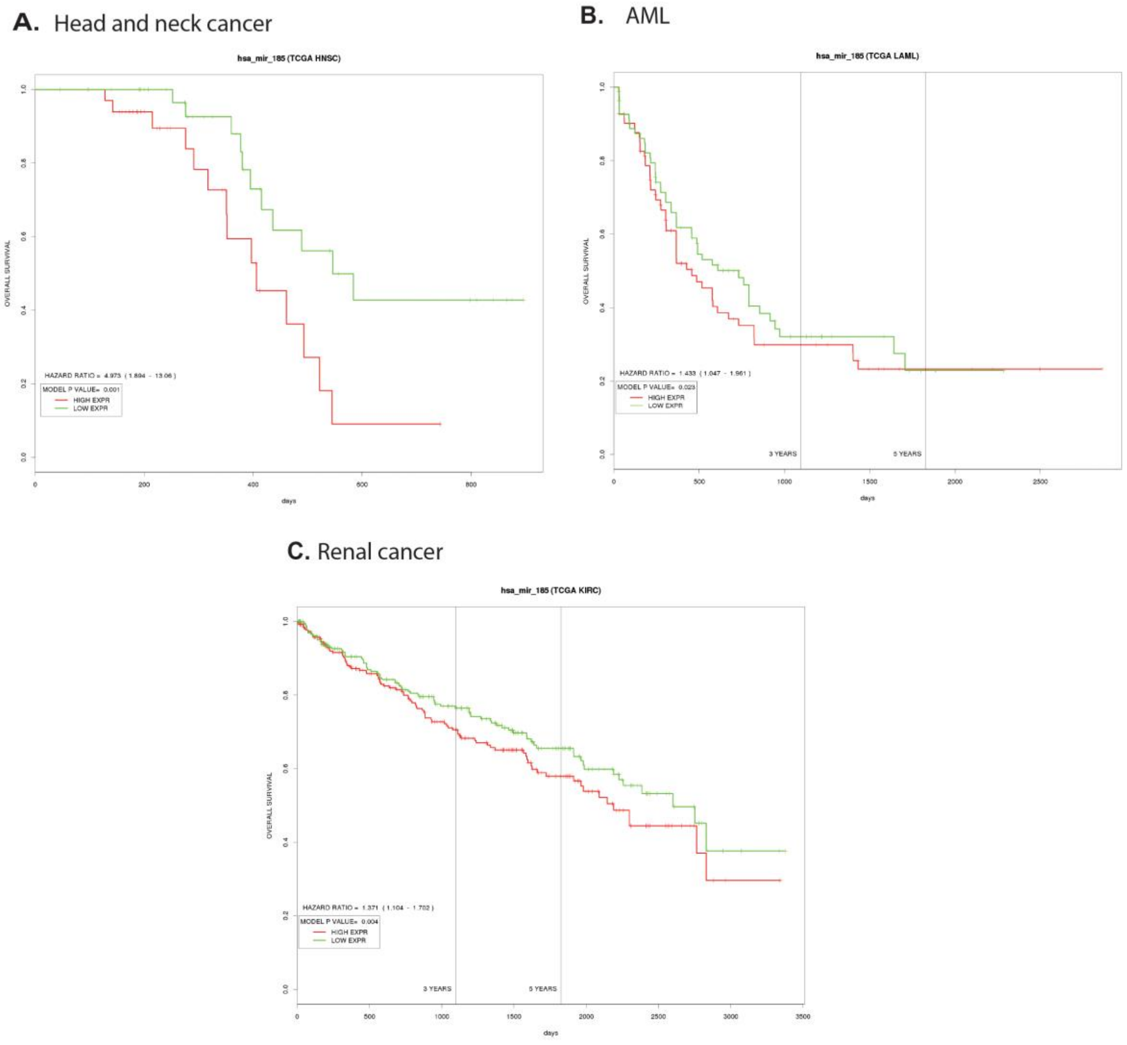

Supplementary Figure 2

The PROGmiR tool was utilized to identify a correlation between miR-185 expression and overall survival of 16 different cancer types. High miR-185 expression was correlated with poor prognosis for head and neck cancer, acute myeloid leukemia, and renal cancer.
